# Detection of *erbB2 *copy number variations in plasma of patients with esophageal carcinoma

**DOI:** 10.1186/1471-2407-11-126

**Published:** 2011-04-11

**Authors:** Immacolata Andolfo, Giuseppe Petrosino, Loredana Vecchione, Pasqualino De Antonellis, Mario Capasso, Donatella Montanaro, Marica Gemei, Giancarlo Troncone, Achille Iolascon, Michele Orditura, Fortunato Ciardiello, Fernando De Vita, Massimo Zollo

**Affiliations:** 1 CEINGE, Centro di Ingegneria Genetica e Biotecnologia Avanzate, Via Gaetano Salvatore 486, 80145, Naples, Italy; 2 Dipartimento di Biochimica e Biotecnologie Mediche, Università degli Studi di Napoli "Federico II", Naples, Italy; 3 Dipartimento Medico-Chirurgico di Internistica Clinica e Sperimentale "F. Magrassi e A. Lanzara" SUN, Seconda Università degli Studi, Naples, Italy

**Keywords:** esophageal carcinoma, cell-free DNA, *erbB2 *copy number variation, prognostic marker, CTCs

## Abstract

**Background:**

Mortality is high in patients with esophageal carcinoma as tumors are rarely detected before the disease has progressed to an advanced stage. Here, we sought to isolate cell-free DNA released into the plasma of patients with esophageal carcinoma, to analyze copy number variations of marker genes in the search for early detection of tumor progression.

**Methods:**

Plasma of 41 patients with esophageal carcinoma was prospectively collected before tumor resection and chemotherapy. Our dataset resulted heterogeneous for clinical data, resembling the characteristics of the tumor. DNA from the plasma was extracted to analyze copy number variations of the *erbB2 *gene using real-time PCR assays.

**Results:**

The real-time PCR assays for *erbB2 *gene showed significant (*P *= 0.001) copy number variations in the plasma of patients with esophageal carcinoma, as compared to healthy controls with high sensitivity (80%) and specificity (95%). These variations in *erbB2 *were negatively correlated to the progression free survival of these patients (*P *= 0.03), and revealed a further risk category stratification of patients with low VEGF expression levels.

**Conclusion:**

The copy number variation of *erbB2 *gene from plasma can be used as prognostic marker for early detection of patients at risk of worse clinical outcome in esophageal cancer.

## Background

Esophageal carcinoma (EC) is the eighth most common cancer and the sixth leading cause of cancer-related death worldwide. In European Union, in 2006, were estimated 25.000 cases per years in men and 8.300 in women [1]. In Italy, data from Italian Association for Tumors Registry (AIRTIUM) accounts in 2003, 2.195 cases with 40 new cases per years in Naples (see: http://www.registri-tumori.it/cms/). The mortality associated with EC is high because tumors are rarely detected before the disease has progressed to an advanced stage. Even when the primary tumor is resectable, the overall 5-year survival rate is under 10% [2]. The stage at which EC is detected is the most important factor in determining prognosis (classified according to the T, N, M system). The rate of lymph node metastasis in both squamous cell carcinoma and adenocarcinoma, the two main histological types of EC, is related to the depth of invasion [3-5]. The majority of ECs that present with symptoms have already invaded the *muscularis propria *(T3) and have already spread to local lymph nodes (N1); this is the reason for the poor prognosis.

New approaches to early detection and monitoring of the course of therapy would benefit the clinical management of patients with EC. Several prognostic factors are currently accepted for clinical use, such as nodal status and tumor stage; however, the disease status and clinicopathological conditions cannot unequivocally identify which patients are at low or high risk for disease recurrence [2,6]. Therefore, there remains the need to identify better prognostic markers that can be used with biological fluids [7,8].

The human epidermal growth factor receptor (*erbB2*) oncogene encodes a transmembrane tyrosine kinase receptor that has evolved as a major classifier of invasive breast cancer and a target of therapy for the disease [9]. However, the role of *erbB2 *in EC is still controversial. Some studies have seen erbB2 over-expression in 20% to 60% of ECs, with this wide range indicating that the differences might depend on tumor stage or histology, or on the interpretation of the immunohistochemistry results. Another study underlined the lack of a prognostic impact of *erbB2 *amplification in primary ECs [10]. An additionally study showed gene-specific quantitative PCR amplification of the *erbB2 *gene in tumor cells from lymph nodes and bone marrow from 98 patients with EC [11]. In 50% of 17q12.21 gains, the *erbB2 *gene was gained in tumor cells from both lymph nodes and bone marrow. Interestingly, only a gain of *erbB2*, and not of 17q12.21, was indicative of poor patient survival, suggesting that *erbB2 *gains are critical for systemic EC. While a gain in *erbB2 *in a single disseminated cancer cell has been shown to be an important risk factor in multivariate analysis, *erbB2 *amplification in primary tumors was not associated with poor survival both in a group of patients that were analyzed for disseminated cancer cells, and in a study cohort comprising twice as many patients [11]. Furthermore, *erbB2 *gene amplification has been demonstrated in the esophageal adenocarcinoma histotype, in 39 patients *versus *39 control samples [12].

The formation of new blood vessels (angiogenesis) and lymph vessels (lymphangiogenesis) significantly contributes to malignant growth and metastasis of solid tumors [13-15]. Angiogenesis and lymphangiogenesis are mediated by distinct cytokines and their receptors. The best characterized and most specific cytokines are the vascular endothelial growth factors (VEGF-A, -B, -C, -D and -E) and their receptors (VEGFR-1, -2 and -3) [16,17]. Clinicopathological and experimental studies on VEGF-C/D expression in esophageal squamous cell specimens and in esophageal adenocarcinoma specimens have been published [18,19]. Studies on esophageal squamous carcinoma have resulted in a relatively consistent correlation of growth-factor expression to tumor progression and lymphatic spread. A recent study reported up-regulation of serum VEGF-C in esophageal squamous cell carcinoma, a finding that parallels VEGF-C expression in tissue specimens [20]. They also correlated serum levels of VEGF-C with the presence of lymph node metastasis, and concluded that VEGF-C up-regulation did not arise from platelets or white blood cells.

Many studies have indicated that soluble DNA from tumors can be detected in the serum and plasma of patients with cancers; alterations in both microsatellites and amplification of oncogenes corresponding to the lesions in tumors have been identified in the serum and plasma of patients with several cancers [21-27]. Analysis of DNA from plasma represents a non-invasive method for detection of circulating tumor cells (CTCs) or their DNA released from the primary tumor into the plasma/serum [21-26]. However, a question that remains debatable is whether this DNA is released from primary dissociating tumor cells or from cells that invade the blood. CTCs have been shown to be a critical link between the primary cancers and metastatic disease, which continues to be the leading cause of death for most malignancies [28]. A sensitive and specific system for quantification of CTCs and their free DNA would thus be a useful diagnostic tool in EC, for monitoring the dissemination of tumor cells into the peripheral serum. As copy-number variations (CN) and over-expression are among the major genomic aberrations in EC pathogenesis, and as the *erbB2 *gene has been found both in circulating tumor cells from lymph nodes and bone marrow of EC patients, here we studied *erbB2 *CN variations in the free DNA from plasma of patients with EC [29]. We show that sensitive and fast real-time PCR technology can indeed detect *erbB2 *CN variations in the free DNA from plasma of EC patients collected before tumors resection and chemotherapy. We also show that *erbB2 *CN variations correlates to VEGF levels and that together can be used as predictive markers for worse clinical outcome.

## Methods

### Sample collection

The present study included 41 patients (men and women; aged 52-74 years) with EC, as diagnosed by the Dipartimento Medico-Chirurgico di Internistica Clinica e Sperimentale "F. Magrassi e A. Lanzara" SUN, Seconda Università degli Studi, Naples, Italy. The ethics committee of the SUN approved this study, and informed written consent was obtained from all of the patients before inclusion in the study. The clinicopathological characteristics of the EC patients are described in Additional file 1: Supplemental Table S1. Thirty-four peripheral EDTA blood samples from 34 healthy volunteers formed the negative control group. The peripheral blood of both cases and controls was centrifuged at 2850 × *g *for 10 min at 4°C, and the supernatant (plasma) was stored at -80°C until analysis. The time lag between blood collection and plasma processing was at most of four hours for both EC patients and healthy controls.

All of the plasma samples from the patients with EC included in the present study were collected before surgical tumor resection and before chemotherapy treatment.

### DNA preparation from plasma

A total of 500 μL of plasma was treated with 1 mg mL^-1 ^proteinase K (GIBCO) and 10% SDS (Sigma Aldrich) for 1 h at 65°C. These samples were then heated to 95°C for 10 min, to inactivate the proteinase K. The DNA in the samples was then phenol extracted and ethanol precipitated. After centrifugation at 6000 × *g *for 15 min at 4°C, the DNA pellets were dissolved in 30 μl sterile water.

### Cloning of the real-time PCR amplification products of erbB2 and β-actin for copy number analysis

Following PCR amplification, the real-time PCR amplification products for *erbB2 *and *β-actin *were cloned separately into the pcr 2.1 vector, using the original TA cloning system (Invitrogen). Briefly, fragments of the *erbB2 *and *β-actin *genes were amplified from genomic DNA of healthy control peripheral blood. The PCR primers and cycling conditions were the same as those used for real-time PCR of the DNA from the EC patients. After the cloning into the pcr 2.1 vector, the erbB2 and β-actin plasmids were prepared at a concentration of 100 ng/μl.

### Determination of ErbB2 copy number variations

#### Real-time PCR

Real-time quantitative PCR (Syber Green method) was performed using standard protocols with an Applied Biosystems ABI PRISM 7900HT Sequence Detection system. Briefly, 100 ng DNA was added to 12.5 μl of SYBR-green PCR master mix (Applied Biosystems), with 600 nM of each primer, and water to 25 μl. The reactions were amplified with a single step of 2 min at 50°C, and 5 min at 95°C, and then for 40 cycles of 5 s at 95°C, and 1 min at 60°C. The thermal denaturation protocol was run at the end of the PCR to determine the number of products that were present in the reactions. All of the reactions were run in triplicate and included non-template controls for each gene. The amount of each gene was normalized to *β-actin *as the reference gene. The real-time PCR primers for each gene were designed using the Primer Express software, version 2.0 (Applied Biosystems), with a Tm of 60°C and a primer length of between 18 nt and 25 nt. We used a standard analysis to calculate the amplification of the *erbB2 *genes by the 2^-DCt method as described previously [30]. The real-time PCR was performed twice for each sample, and we used the mean value of these two independently data points.

### Calibration curves

For each assay, we prepared a reference calibration curve as described previously, [31] which containing a nine-concentration titration series representing the *erbB2 *and *β-actin *genes at 10-fold dilutions from 1.0 ng/μl to 1.0 × 10^-8 ^ng/μl (see Additional file 2: Supplemental Figure S1). Each calibration curve was produced in triplicate. We tested the reproducibility of the calibration curves for *erbB2 *and *β-actin *genes determination according to the slopes and the correlation coefficients of the experimental fittings of the calibration curves from each experiment. The mean slopes of the calibration curves for the two genes were similar: -3.285 for *erbB2 *and -3.036 for *β-actin*. The mean correlation coefficients of the curve fitting were 0.989 and 0.994, respectively. We here, showed three representative slopes for β-actin and erbB2 with the standard errors: slopes β-actin: -3.122; -2.772; -3.215, standard deviation is 0.234 and standard error is 0.135; slopes erbB2: -3,263; -3,232; -3,360, standard deviation is 0,066 and standard error 0.039. A slope of -3.3 +/- 10% reflects an efficiency of 100% +/- 10% of the PCR reaction [32]. For this reason, the slopes for the two genes are similar (*p values *= 0,150). We calculates these gene CN of the patients using the Ct values of the vector and calculating the CN vector according to the formula [31,33,34]:


This formula takes into account 6.022 × 10^23 ^(molecules/mole) that is the Avogadro's number and 660 Da that is the average weight of a single base pair. The non-integer results were treated on the basis of the cut-off CN >2 and CN ≤2 and they were rounded off, particularly, the values were rounded down if the decimal value was between 0 and 4; while they were rounded up if the decimal value was between 5 and 9.

The primers for the *erbB2 *gene were:

*erbB2 *F: TATGCAGGGCTGACGTAGTGC

*erbB2 *R: AATGTGTGCCACGAAACTGCT

The primers for the *β-actin *gene were:

β-actin F: CCTCACCCTGAAGTACCCCA,

β-actin R: TCGTCCCAGTTGGTGACGAT.

### Cell sorting of circulating tumor cells by flow cytometry

Whole peripheral blood samples from six EC patients were centrifuged at 2850 × *g *for 10 min at 4°C, and the plasma was removed. The resulting cell pellet was suspended by dilution with phosphate-buffered saline (PBS) and then centrifuged at 1700 × *g *for 30 min at 4°C, to separate mononuclear cells. Then, the mononuclear cells were stained at 4°C and avoiding light exposure, with: cytokeratins CK8-, CK18-, and CK19-FITC (monoclonal antibody A45-B/B3; Micromet, Munich, Germany) 1:30; CD326-APC (EpCAM, Becton Dickinson) 1:10; and CD45-PerCP (Becton Dickinson) 1:10. The samples were then washed with 2 ml PBS with 2% fetal bovine serum, and centrifuged at 800 × g for 3 min at 4°C. The supernatant was removed and the cell pellets were suspended as before (2 ml PBS with 2% fetal bovine serum), and filtered with 30 μm filcons (Becton Dickinson) before analysis by flow cytometry. The flow cytometry gating strategies included: a first gate based on physical parameters forward scatter (FSC) *versus *side scatter (SSC), to eliminate cell debris and dead cells. The leukocyte population was excluded with a second gate in the CD45 *versus *SSC dot plot. The CTCs were selected by a gate based on cytokeratin CK8, CK18 and CK19/CD326-positive cells. These cells were purified by cell sorting with a BD FACS Aria instrument, and collected in a 2 ml tube.

### Immunohistochemistry

One or two representative tumor blocks from each patient were examined by immunohistochemistry. We analyzed erbB2 for 14 EC tissue samples with an anti-erbB2 antibody (1:500; DakoCytomation). The unmasking was performed in 10 mM citrate buffer, pH 6, for 45 min at 97°C. Blocking was performed with BSA 2,5%, normal goat serum 0.05%, PBS 1%, Tween 0,5% for 1 h at room temperature. The signal was revealed according to DAKO kits, for 15 min (each, for biotin and streptavidin), at room temperature. DAB was from DakoCytomation, and the slides were mounted and examined under a Leica DC500 compound microscope (Nussloch, Germany).

ErbB2 immunohistochemical staining in EC samples was scored according to *Kuwabara et al.*, [35], who adopted the same criteria previously validated for breast cancer; namely: no staining or weak staining in fewer than 10% of the tumor cells (-); weak staining in part of the membrane in more than 10% of the tumor cells (+); complete staining in part of the membrane with weak or moderate intensity in more than 10% of the tumor cells, (++); strong staining in more than 10% of the tumor cells, (+++).

### VEGF ELISA assay

The levels of the VEGF protein in the plasma used for *erbB2 *amplifications from 41 EC patients were determined using a commercially available sandwich enzyme immunoassay kit (Endogen VEGF ELISA kit, Cambridge, USA). All sample were assayed in duplicate.

### Statistical analysis

All of the data are presented as medians ± standard error. Statistical significance was calculated using the Mann-Whitney test. Raw real-time PCR data for *erbB2 *amplification in the plasma from healthy controls and patients with EC were normalized using the *β-actin *amplification values. The *erbB2 *CN values were divided into two groups, as "CN ≥2" and "CN <2". Potential associations between clinicopathological variables and *erbB2 *CN were analyzed by Pearson's Chi-Squared. Kaplan-Meier survival curves were constructed for *erbB2 *and VEGF low and high level groups, as well as for the clinicophathological variables available in our dataset. Differences in survival between the groups were tested for statistical significance by log-rank tests. Progression free survival time was measured from the date of registration in the study to the date of progression or last follow-up visit. The patients derived all from one clinic and were all of Caucasian origin. *P *< 0.05 was considered as statistically significant.

Receiver operating characteristic (ROC) curves and the area under the ROC curves were generated to calculate the specificity and sensitivity of *erbB2 *CN variation in the healthy controls and the EC patients.

## Results

### Detection of erbB2 copy number variations in DNA from plasma of patients with esophageal carcinoma

**Figure 1:**
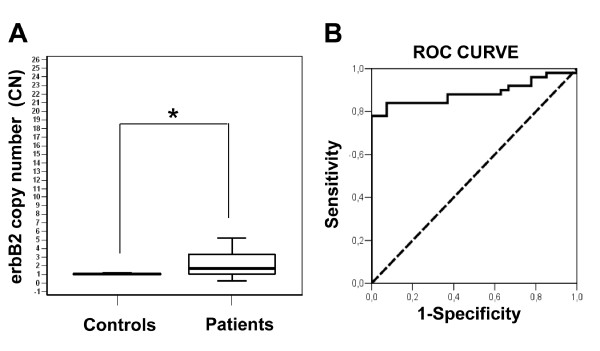
***ErbB2 *copy number variations in DNA in plasma from patients with esophageal carcinoma *versus *healthy controls and relative ROC curve analysis of the assay**. (**A**) Box plot showing the ranges of *erbB2 *CN in DNA from the plasma of patients with EC (n.41) with respect to healthy controls (n.34). *P = 0.001. Horizontal bars in the box represent the medians of CN which is equal to 2 for patients and 1 for controls. (**B**) Receiver operating characteristic (ROC) curves (solid line; AUC, 0.95) were generated to compare healthy controls with EC patients for *erbB2 *with the null hypothesis (dashed line; AUC, 0.5; *P *= 1.39 × 10^-11^). The assay showed 80% sensibility and 95% specificity. Data are representative of three independent experiments.

Real-time PCR with CN analysis was used for the analysis of DNA from the plasma of 41 patients with EC, before their surgical resection and chemotherapy treatment. Here, we also analyzed the CN variations for *erbB2 *in 34 healthy controls. As shown in Figure 1A, the *erbB2 *CNs were significantly higher in the plasma samples from the 41 EC patients, with respect to the 34 healthy control subjects (*P *= 0.001). In particular, 24 EC patients had CN ≤2 (58.5%), while 17 EC patients had CN >2 (41.4%) with a median CN of 2 and a standard deviation of 5.02. The 34 healthy control subjects showed a median of *erbB2 *CN of 1, with a standard deviation of 0.16. The representative calibration curves for the *erbB2 *and *β-actin *genes used for the CN variation analysis are shown in the Additional file 2: Supplemental figure S1 (Figure S1).

We also evaluated the CN data of *erbB2 *by real-time PCR using the ROC curves, which provide an analysis of the sensitivity and specificity of the assay. The ROC curves were constructed for the *erbB2 *CN in plasma, examining the 41 EC patients and 34 healthy controls. As shown in Figure 1B, in this sample set, *erbB2 *had an area under the curve (AUC) of 0.95, and differed significantly from that of a chance result (AUC: 0.5) (*P *= 1.39 × 10^-11^). For distinguishing patients with EC from healthy controls, *erbB2 *had an 80% sensibility, and a 95% specificity (Figure 1B).

The association of the *erbB2 *CN variations with the clinicopathological characteristics of the EC patients are summarized in the Additional file 1: Supplemental table S1. The association of *erbB2 *CN and the clinical features of the esophageal carcinoma were also assessed by the Chi-Squared Test. There were no significant statistical direct correlations between *erbB2 *CN and these clinical features, except for tumor location when associated with *erbB2 *CN >2 (*P *= 0.05). Despite the small number of patients, our dataset comprises all the characteristic of the tumor as regard T stage, N status, tumor grading, tumor location and histology, see Additional file 1: Supplemental table S1 (tab.S1). All CT values of real time PCR assay for EC patients and healthy controls subjects were showed in the Additional file 3: Supplemental table S2 (tab.S2).

### ErbB2 copy number variations predict survival rates in esophageal carcinoma patients

**Figure 2:**
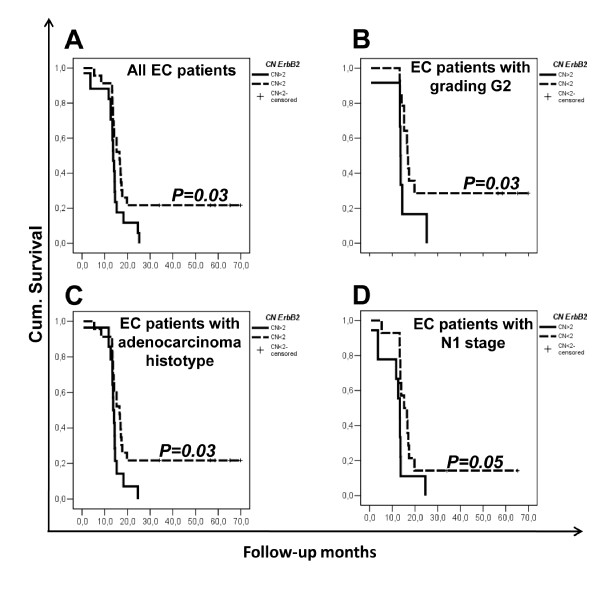
**Kaplan-Meier survival curves for e*rbB2 *copy number variations in plasma from patients with esophageal carcinoma according to clinical characteristics**. (**A**) All patients with EC; (**B**) EC patients with tumor grading G2; (**C**) EC patients with adenocarcinoma histology; (**D**) EC patients with N1 stage disease. The patients are divided according to *erbB2 *CN ≤2 (solid line) and *erbB2 *CN >2 (dashed line) subgroups. Data are representative of three independent experiments.

We used Kaplan-Meier analyses to look for correlations between the *erbB2 *CN variations and EC patient progression-free survival. The survival curves for the EC patients were divided according to *erbB2 *CN ≤2 and *erbB2 *CN >2, as illustrated in Figure 2 on the basis of CN median of EC patients. We also tried to divide the patients into lose (CN <1), normal (2 ≥ CN ≥1) and gain (CN ≥ 2) of *erbB2*, 6, 14 and 21 patients respectively, and by using Kaplan-Meier analyses we did not reach a statistical significance but we saw the same tendency as for the survival analysis with cut-off 2 (Additional file 2: Supplemental Figure S1 C). The number of patients here analyzed is too small to be divided into three groups, for this reason we analyzed all the data with the CN cut-off value of 2. *ErbB2 *CN >2 was significantly negatively correlated to the survival rates of these EC patients (*P *= 0.03; Figure 2A).

In further analyses according to the tumor grading, the EC patients with a tumor grading G2 can be further stratified according to *erbB2 *CN ≤2 and *erbB2 *CN >2, which were again significantly negatively correlated to the survival rates of these EC patients (*P *= 0.03; Figure 2B). These analyses for the EC patients with tumor gradings G1 and G3 did not show any statistically significant effects on their survival rates due to the small numbers of patients in these subgroups (data not shown).

Our dataset also showed an heterogeneous histological composition when stratified accordingly to the histotype. Here, *erbB2 *CN variations were significantly correlated to the survival rates of the adenocarcinoma cases (*P *= 0.03; Figure 2C). Similarly, we analyzed the correlations between the N status and survival rates: *erbB2 *CN >2 in the N1 subgroup was significantly negatively correlated to the survival rates of these EC patients (*P *= 0.05, Figure 2D). Again, these analysis for the EC patients with an N status of N2 and N3 did not show any statistical significance for the survival rates due to the small numbers of patients in these subgroups (data not shown).

### Correlations of erbB2 copy number variations with VEGF plasma levels

**Figure 3:**
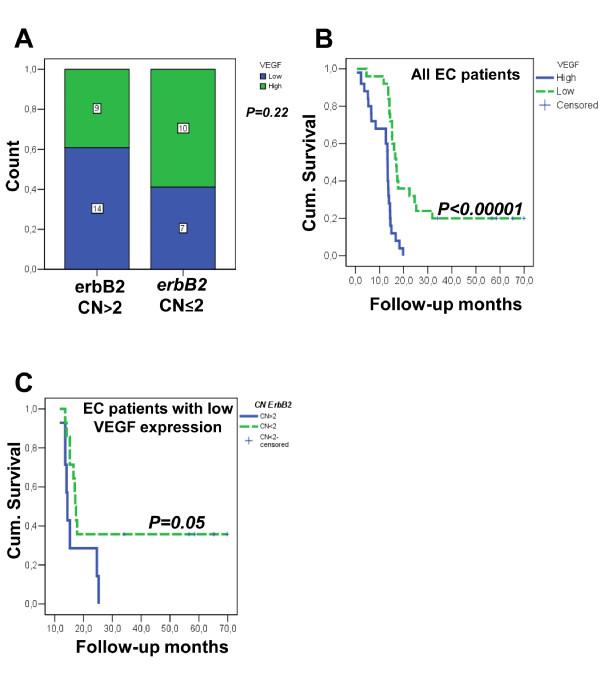
***ErbB2 *copy number variations in plasma from patients with esophageal carcinoma according to VEGF plasma levels**. (**A**) Histogram showing lack of significant correlation between *erbB2 *CN variations and VEGF plasma levels in all patients with EC (n. 41), as indicated. The numbers in the bars show the EC patients in each sub-group. (**B, C**). Kaplan-Meier survival curves for all patients with EC according to low and high plasma levels of VEGF **(B)**, and for patients with EC and low plasma levels of VEGF according to *erbB2 *CN ≤2 (solid blu line) and *erbB2 *CN >2 (dashed green line). Data are representative of three independent experiments.

**Figure 4:**
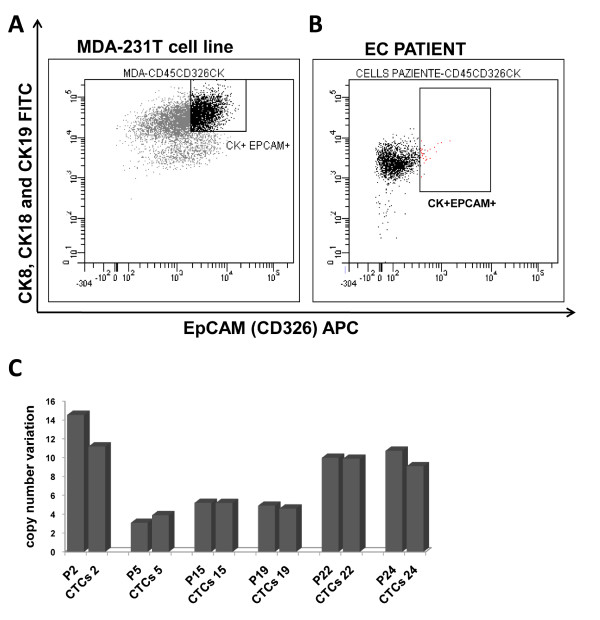
**Isolation of CTCs using fluorescence-activated cell sorting**. (**A, B**) Scatter plots for gating of cells positive to CK8, CK18, CK19 and EpCAM in the MDA-231T breast cancer cell line (A; positive control) and from the blood of a representative patient with EC (B). (**C**) Histogram showing *erbB2 *CN variations from sorting CTCs of patients with EC compared to the CN of DNA from plasma of the same patients. Data are representative of three independent experiments.

We then tested *erbB2 *CN for possible associations with VEGF protein levels in the plasma from the same EC patients (Figure 3). The VEGF levels data were available in our tissue/serum databank collection, and have been previously published [36]. The *erbB2 *CN variations did not show any significant direct associations with VEGF levels in the plasma (Figure 4A). However, when the EC patients were stratified into the low and high VEGF groups, as shown in Figure 4B, high VEGF levels in the plasma of those EC patients were significantly negatively correlated to their survival rates (*P *< 0.00001). In a further Kaplan-Meier analysis, we also looked at the influence of *erbB2 *CN variations in these EC patients with low and high VEGF levels: an *erbB2 *CN >2 was significantly negatively correlated to the survival rate of the EC patients with low VEGF expression (*P *= 0.05; Figure 3C). Altogether, these results show that *erbB2 *CN >2 status and low VEGF levels in the plasma of these EC patients appear to be useful to stratify a subgroup of patients with worse survival rates.

### Isolation of circulating tumor cells from peripheral blood of esophageal carcinoma patients

To answer the question of whether this DNA in the plasma of these EC patients was derived from CTCs, we isolated these cells from these patients. Indeed, we detected CTCs that were disseminated in the peripheral blood in six of these patients with EC. The CTCs were isolated from the peripheral blood of these EC patients by sorting the cells negatively for CD45 (a specific antigen for lymphocytes) and by a gate based on epithelial cytoskeleton components: the cytokeratins CK8, CK18 and CK19, and CD326 (EpCAM) (see Methods). Both, the cytokeratins (CK8, CK18 and CK19) and the antigen EpCAM have been used to identified epithelial cell adhesion molecules, as previously used by *Stoecklein et al. *as markers to isolate CTCs from lymph nodes and bone marrow of patients with EC [11]. As previously described, [37] we used the MDA-231T breast cell line as the positive control (Figure 4A). Here we show that we were only able to successfully isolated positive cells from the blood samples from the EC patients (about 0.05%-0.1% of the 2.0 × 10^6 ^cells analyzed; Figure 4B), from which we prepared genomic DNA for the determination of *erbB2 *CN. This analysis was performed in all of the other five samples analyzed, with similar results (data not shown). The peripheral blood of the healthy controls was analyzed in the same way, but did not show any cell population positive for CK8, CK18 and CK19 and EpCAM, and negative for CD45 (data not shown). The analyses of this DNA extracted from these CTCs of the EC patients showed that the *erbB2 *CN was in the same range (CN >2) as for the DNA isolated from the plasma of the same (six) EC patients (Figure 4C). The comparison between the CN values from plasma and from CTCs was showed in Additional file 4: Supplemental table S3 (tab.S3).

### ErbB2 protein expression in tumor tissue

We additionally performed immunohistochemistry analysis for erbB2 protein staining (in parallel sections) in 13 esophageal primary tumor tissue samples, four of the tissues of which were included in the plasma DNA dataset previously analyzed. Some tissue staining examples are shown in the Additional file 5: Supplemental Figure S2. Strong expression of erbB2 was showed for the case code 1 patient (score +++), with intermediate expression of erbB2 for the case code 2 patient (score ++) and low expression for the case code 3 patient (score +) (the Additional file 5: Supplemental Figure S2A). Control experiments showed weak staining of erbB2 in proliferative epithelial tissues of healthy esophageal mucosa (Additional file 5: Supplemental Figure S2 B).

## Discussion

In clinical practice, the diagnosis and the staging of EC is mainly based on the use of morphological and functional clinical examinations. To date, serum-specific EC markers do not exist, which is in contrast with the situation for other tumors of the gastrointestinal tract. Examples here, can be seen for gastric cancer, where Ca.19.9 has important role in diagnosis. At this time, we can conclude that already identified single biomarkers show a lack of sufficient sensitivity and specificity in EC, and that it is likely that multiple markers will be needed simultaneously to address the diagnosis and prognosis of EC [3].

The relationship between erbB2 over-expression and responses to treatment has not yet been established for EC. New perspectives have been emerging, such as for instance from the TOGA trial, that showed efficacy results from a phase III study of trastuzumab (Herceptin^®^), a monoclonal antibody against erbB2: when given with chemotherapy, survival benefits were seen for patients with erbB2-positive early and metastatic gastric cancer [9]. Thus, the data in this TOGA trial showed that trastuzumab with chemotherapy is superior to chemotherapy alone, with median overall survival significantly improved with trastuzumab compared to chemotherapy alone: 13.5 *versus *11.1 months, respectively. Trastuzumab is therefore a new and well-tolerated treatment for erbB2-positive gastrointestinal cancer [38].

VEGF is an additional marker, which is over-expressed in about 30% to 60% of esophageal tumors. High serum levels of VEGF correlate with advanced stages and poor overall survival in patients that receive curative surgery [39]. Several studies have shown that VEGF-C over-expression correlates with depth of tumor invasion, lymphatic invasion and lymph node metastasis.

CTCs and free DNA in plasma have been analyzed in many tumors, such as breast, colorectal, lung and renal carcinomas. These have been shown to be crucial links between primary cancers and those that reach a metastatic stage, which represent the leading cause of death for most malignancies [40-46]. Our study has evaluated the evolving model of detection of tumor marker CN in DNA from plasma of EC patients. We have here identified *erbB2 *CN variations in the cell-free DNA of 41 patients with EC in comparison with 34 healthy controls. Our results are in agreement with the study of *Chiang PW. et al.*, that showed *erbB2 *amplification in 39 plasma from esophageal carcinoma patients with only adenocarcinoma histotype. The system of detection of *erbB2 *in DNA from plasma by real-time PCR is innovative and with the validation in a larger cohort of patients can be used for monitoring the assessment of the extent of tumor dissemination and the potential development of distant metastasis. This method uses an easier and not invasive peripheral blood sample in respect with the FISH assay *(PathVysion FISH assay*) performed on tumor biopsies actually approved by the US Food and Drug Administration (FDA) for determining the eligibility for Herceptin clinical treatment in breast carcinoma with *erbB2 *amplification.

We have further analyzed the *erbB2 *CN from CTCs isolated from patients with EC and we have demonstrate that it is similar to the DNA isolated from the plasma of the same patients. These data indicate that in the healthy controls, the DNA that we extracted was derived from blood cells (mainly lymphocytes and granulocytes); instead, in the EC patients, the free DNA in the plasma is mainly derived from CTCs (see Additional file 6: Supplemental Table S4, for estimation of the DNA content in plasma). At this time, a question remains over the origin of these CTCs: Are those cells derived from primary tumors or from cells that will create a metastases foci? We cannot at present exclude that this DNA derived from the primary tumor cells because of our lack of DNA from the primary tumors in this study. However, our data related to the *erbB2 *CN, shown for this cohort analysis, is in agreement with another study that has shown *erbB2 *amplification in CTCs isolated from lymph nodes and bone marrow in 50% of EC patients [11]. This study demonstrated that the rate of *erbB2 *amplification is higher in CTCs than in the primary tumors, where the rates of amplification were estimated at about 10% [10,47]. These data are in agreement with the observation that *erbB2 *CN variations are a later phenomenon in EC that is mainly associated with the circulating tumor cells. Our data additionally demonstrate that the CTCs isolated from the peripheral blood of these EC patients have a similar type of *erbB2 *amplification to that seen by *Stoecklein et al. *in CTCs isolated from bone marrow and lymph nodes [11]. The present study underlines that the detection of cell-free DNA from plasma through the taking of a non-invasive blood sample can provide relevant information on the disease status of patients with EC. It is important to underline that despite the small number of EC patients (n.41) included in our cohort, the incidence of this tumor is of 2195 cases for year in Italy with 40 cases in Naples and a raw rate (number of cases per one hundred thousand of inhabitants for year) of 2 in southern Italy (data from Italian Association tumors register AIRTUM 2000-2003; see: http://www.registri-tumori.it/cms/). Furthermore, our dataset is heterogeneous for composition resembling all the clinical characteristics of the esophageal carcinoma.

We have shown here a correlation between *erbB2 *CN from plasma and progression-free survival in patients with EC with 70 months (5,8 years) of follow-up. The EC patients were divided into two subgroup on the basis of CN median (cut-off CN = 2), other separation in subgroups were made but they did not shown correlation to progression-free survival for the small number of patients here analyzed (see the Additional file 2: Supplemental Figure S1 C). The EC patients with *erbB2 *CN ≤2 showed a better survival with respect to those with CN >2 *erbB2*. These data demonstrate that the detection of *erbB2 *CN in the plasma of EC patients can predict the survival rates of patients before the use of clinical resection strategies. These data emphasize the results obtained by *Mimura et al.*, that found an association only between the survival rate and the *erbB2 *gene amplification (7 positive patients *versus *59 negative patients) detected by fluorescence in situ hybridization (FISH) method and not between the survival rate and positive or negative erbB2 staining detected by immunohistochemistry assay [46]. Furthermore, *Kuwabara et al.*, also demonstrated the correspondence between gene amplification (analyzed by FISH) and protein expression (analyzed by immunohistochemistry) of erbB2, underlining that gene amplification is an indicator of poor prognosis in esophageal carcinoma [35].

In particular, we found that an *erbB2 *CN >2 was correlated to a worst survival rate in the adenocarcinoma histotype. Of note, esophageal adenocarcinoma has been shown to have increased in western countries over the last half century, and especially in European Caucasian white men, although esophageal squamous cell carcinoma remains the dominant type of EC in both western and Asian countries [47-49]. The rapid increase of this adenocarcinoma histotype has been attributed to an increased prevalence of gastroesophageal reflux disease and obesity [48-50].

Furthermore, our dataset confirms the importance of N status evaluation through our Kaplan-Meier analysis. When divided according to N status, an *erbB2 *CN >2 impaired survival rates of patients with an N1 status, confirming the importance of the amplification of the *erbB2 *gene in the prediction of a worse clinical outcome.

We also confirmed the importance of the VEGF expression in our dataset, demonstrating that high VEGF expression impairs the survival rate of these EC patients. Moreover, we have identified a new EC category risk: low VEGF expression and *erbB2 *CN >2. These data are of importance because of the potential new therapies, anti-erbB2, that might be applied to EC patients who will not derive any benefit from anti-VEGF treatment.

## Conclusion

The use of these methods to detect DNA in the plasma of such EC patients should greatly benefit the "early detection" phase before tumor resection and chemotherapy, and should enhance the importance for changes in therapies for those low VEGF-positive EC patients. The validation of the *erbB2 *detection in a large scale population thus represents a new prognostic marker that can be used as a sign of EC early warning, and for the prediction of the disease progression. Thus, inhibition of *erbB2 *activity might represent a new avenue for successful inhibition of potential metastasis formation, and represents a new therapeutic target for future personalized cancer therapies.

These data indicate the association between *erbB2 *CN variations and progression-free survival in these patients with EC. As previously said, the number of patients analyzed here is not sufficient to draw a conclusion on the possible diagnostic application, although we know that our tumors dataset represents a well morphological distribution in the Caucasian population affected by esophageal cancer. At this time, a large study that can be designed to include both EC and other tumors of the gastrointestinal tract, thus will definitively enhancing our methodology and findings.

## Abbreviations

EC: esophageal carcinoma; CN: copy number variations; CTCs: circulating tumor cells.

## Competing interests

The authors declare that they have no competing interests.

## Authors' contributions

IA conducted and designed most of the experiments and participate to the manuscript writing and editing; GP performed the cloning for the real time experiments; LV performed samples collection; PDA performed real time set-up experiments; MO coordinate samples collection and clinical database construction, MC performed statistical analysis; DM performed the immunohistochemistry analyses; MG performed FACS analyses; GT performed the pathological scoring; FC and FDV contributed to the collection of blood samples, clinical follow-up and preparation of the manuscript; MZ design and coordinate the study, then contribute to the final critical review and editing of the manuscript.

All authors read and approved the final manuscript.

## Pre-publication history

The pre-publication history for this paper can be accessed here:

http://www.biomedcentral.com/1471-2407/11/126/prepub

## Supplementary Material

Additional file 1 Supplemental Table S1: Association of erbB2 copy number variations with the clinicopathological features of the patients with esophageal carcinoma. Click here for file

Additional file 2 **Supplemental Figure S1: Calibration curve for *erbB2 *and *β-actin *genes relating cycle threshold to gene copy number**. (**A**) For the *erbB2 *gene CN. Top right, equation of the curve and the relative mean correlation coefficient (R2). (**B**) For the *β-actin *gene CN. Top right, as for (A). (**C**) Kaplan-Meier survival curves for all patients with EC according to lose, normal and gain erbB2 CN. Click here for file

Additional file 3 Supplemental Table S2: CT values of real time PCR of erbB2 and β-actin genes for all patients and controls. Click here for file

Additional File 4 Supplemental Table S3: Copy number variation of CTCs from EC patients compared to copy number variation of DNA from plasma of the same patients. Click here for file

Additional File 5 **Supplemental Figure S2: ErbB2 in esophageal tumor tissues**. (**A**) Patterns of ErbB2 staining with a polyclonal antibody in EC tumor tissues 1, 2 and 3, showing scores of +++, ++ and +, respectively. (**B**) ErbB2 staining as for (A), in healthy esophageal mucosa (CTR). Click here for file

Additional File 6 Supplemental Table S4: Quantization of DNA extracted from plasma of patients and controls. Click here for file
