# Supplementary material for: Detection of erbB2 copy number variations in plasma of patients with esophageal carcinoma
Source: BMC Cancer. 2011 Apr 11;11:126. doi: 10.1186/1471-2407-11-126 (PMC3094322; doi:10.1186/1471-2407-11-126)
Supplement: Additional file 1 — Supplemental Table S1: Association of erbB2 copy number variations with the clinicopathological features of the patients with esophageal carcinoma. [file 1471-2407-11-126-S1.DOC]

| **Features** | **Number of patients** | ***erbB2* copy number (CN)** | | ***P*** |  |
| --- | --- | --- | --- | --- | --- |
|  | **(%)** | **≤2 (%)** | **>2 (%)** |  |  |
|  |  |  |  |  |  |
| **Age (years)** |  |  |  |  |  |
| **>60** | 9 (22) | 5 (20.8) | 4 (23.5) | 0.84 |  |
| **≤60** | 32 (78) | 19 (79.2) | 13 (76.5) |  |  |
| **T stage** |  |  |  |  |  |
| **T1** | 0 (0) |  |  |  |  |
| **T2** | 8 (20) | 4 (16.7) | 4 (25) | 0.81 |  |
| **T3** | 27 (67.5) | 17 (70.8) | 10 (62.5) |  |  |
| **T4** | 5 (12.5) | 3 (12.5) | 2 (12.5) |  |  |
| **N stage** |  |  |  |  |  |
| **N0** | 15 (39.5) | 9 (39.1) | 6 (40) | 0.96 |  |
| **N1** | 23 (60.5) | 14 (60.9) | 9 (60) |  |  |
| **N2** | 2 (3.4) | ND | ND |  |  |
| **Tumor grading** |  |  |  |  |  |
| **G1** | 9 (24.3) | 5 (22.7) | 4 (11.1) | 0.28 |  |
| **G2** | 20 (54.1) | 14 (63.6) | 6 (40) |  |  |
| **G3** | 3 (13.6) | 5 (33.3) | 8 (21.6) |  |  |
| **Tumor location** |  |  |  |  |  |
| **upper** | 7 (17.1) | 6 (25) | 1 (5.9) | 0.05 |  |
| **middle** | 20 (48.8) | 8 (33.3) | 12 (70.6) |  |  |
| **lower** | 14 (34.1) | 10 (41.7) | 4 (23.5) |  |  |
| **Histology** |  |  |  |  |  |
| **squamous** | 26 (63.4) | 13 (54.2) | 13 (76.5) | 0.14 |  |
| **adenocarcinoma** | 15 (36.6) | 11 (45.8) | 4 (23.5) |  |  |
|  |  |  |  |  |  |

**Additional file1: Supplemental Table S1.** Association of *erbB2* copy number variations with the clinicopathological features of the patients with esophageal carcinoma

T and N, the clinical stages evaluated according to the UICC staging criteria.
