# Supplementary figures and images for: Detection of erbB2 copy number variations in plasma of patients with esophageal carcinoma
Source: BMC Cancer. 2011 Apr 11;11:126. doi: 10.1186/1471-2407-11-126 (PMC3094322; doi:10.1186/1471-2407-11-126)

**A**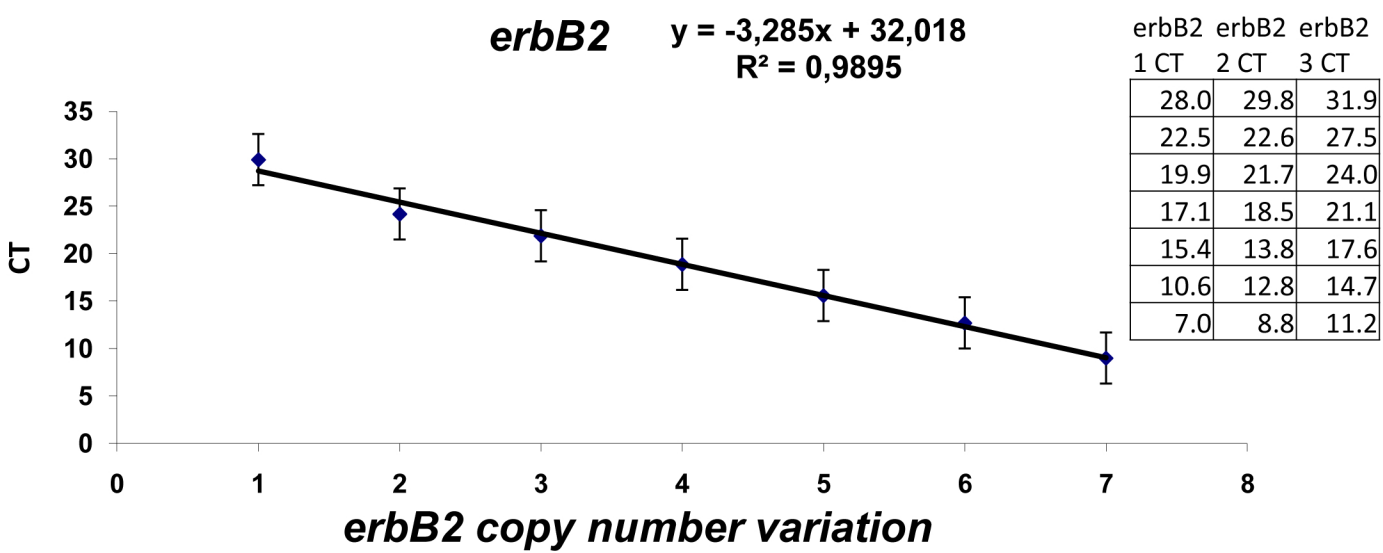**B**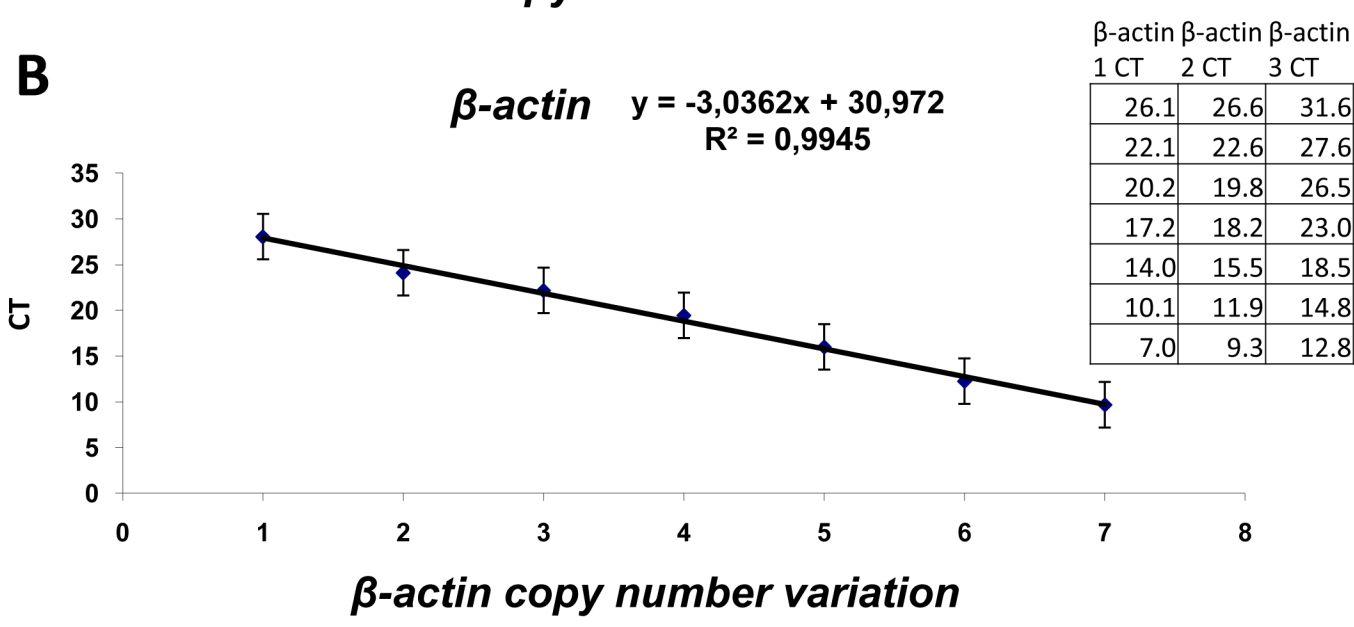**C**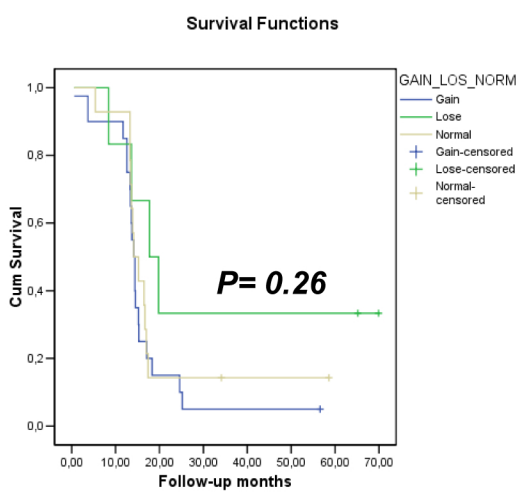

Supplement: Additional file 2 — Supplemental Figure S1: Calibration curve for erbB2 and β-actin genes relating cycle threshold to gene copy number. (A) For the erbB2 gene CN. Top right, equation of the curve and the relative mean correlation coefficient (R2). (B) For the β-actin gene CN. Top right, as for (A). (C) Kaplan-Meier survival curves for all patients with EC according to lose, normal and gain erbB2 CN. [file 1471-2407-11-126-S2.PDF]

**A**

**ErbB2**

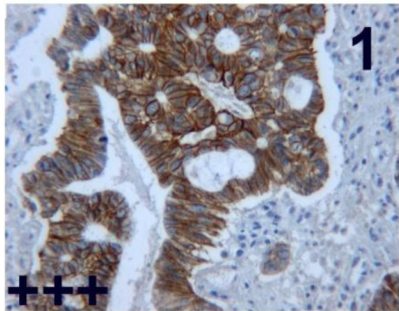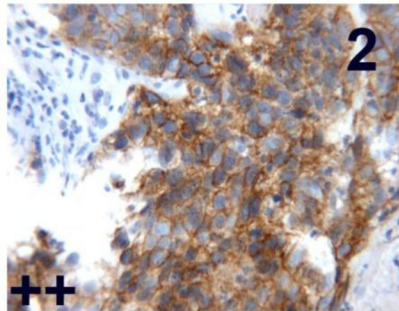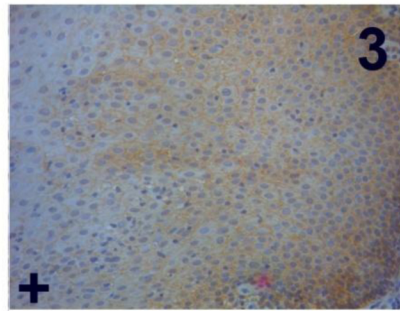

**B**

**ErbB2**

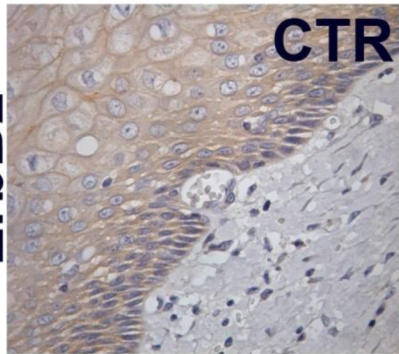

Supplement: Additional File 5 — Supplemental Figure S2: ErbB2 in esophageal tumor tissues. (A) Patterns of ErbB2 staining with a polyclonal antibody in EC tumor tissues 1, 2 and 3, showing scores of +++, ++ and +, respectively. (B) ErbB2 staining as for (A), in healthy esophageal mucosa (CTR). [file 1471-2407-11-126-S5.PDF]
