# Supplementary material for: Detection of erbB2 copy number variations in plasma of patients with esophageal carcinoma
Source: BMC Cancer. 2011 Apr 11;11:126. doi: 10.1186/1471-2407-11-126 (PMC3094322; doi:10.1186/1471-2407-11-126)
Supplement: Additional file 3 — Supplemental Table S2: CT values of real time PCR of erbB2 and β-actin genes for all patients and controls. [file 1471-2407-11-126-S3.DOC]

| **Additional file 3. Supplemental Table S2.** CT values of real time PCR of *erbB2* and *β-actin* genes for all patients and controls | | | | | |  |
| --- | --- | --- | --- | --- | --- | --- |
| ***EC patients code*** | ***CT erbB2*** | ***CT β-actin*** | ***Healthy subjects code*** | ***CT erbB2*** | ***CT β-actin*** |  |
| P1 | 21.94 | 20.46 | e10 | 31.54 | 26.63 |  |
| P2 | 25.97 | 26.01 | e12 | 25.13 | 20.16 |  |
| P3 | 31.77 | 26.45 | e13 | 28.62 | 24.28 |  |
| P4 | 23.54 | 22.50 | e14 | 29.46 | 24.66 |  |
| P5 | 28.54 | 27.04 | e2 | 34.69 | 29.60 |  |
| P6 | 26.84 | 27.55 | e20 | 31.69 | 26.91 |  |
| P7 | 26.80 | 25.02 | e22 | 29.20 | 24.88 |  |
| P8 | 23.70 | 22.45 | e23 | 27.59 | 22.78 |  |
| P9 | 23.28 | 21.80 | e3 | 28.53 | 23.72 |  |
| P10 | 28.98 | 27.29 | e40 | 26.75 | 23.63 |  |
| P11 | 21.79 | 20.40 | e41 | 23.36 | 20.97 |  |
| P12 | 29.51 | 27.62 | e46 | 29.43 | 26.72 |  |
| P13 | 26.50 | 25.38 | e48 | 27.99 | 24.96 |  |
| P14 | 28.57 | 26.85 | e49 | 28.58 | 23.38 |  |
| P15 | 27.71 | 26.64 | e5 | 31.21 | 25.92 |  |
| P16 | 22.51 | 21.11 | e51 | 29.03 | 23.99 |  |
| P17 | 26.57 | 25.02 | e52 | 29.73 | 24.83 |  |
| P18 | 21.80 | 20.64 | e53 | 27.69 | 22.62 |  |
| P19 | 27.50 | 26.48 | e55 | 30.00 | 24.88 |  |
| P20 | 26.43 | 25.27 | e56 | 27.81 | 22.51 |  |
| P22 | 37.16 | 26.94 | e57 | 30.34 | 24.94 |  |
| P24 | 22.98 | 21.16 | e6 | 29.04 | 23.84 |  |
| P25 | 24.21 | 21.98 | e7 | 30.16 | 24.79 |  |
| P26 | 24.28 | 22.87 | e8 | 29.54 | 24.13 |  |
| P27 | 24.73 | 22.87 | f3 | 24.09 | 20.37 |  |
| P28 | 30.16 | 27.35 | f4 | 23.01 | 19.44 |  |
| P29 | 27.63 | 25.78 | f5 | 25.96 | 22.33 |  |
| P30 | 23.69 | 21.59 | f6 | 24.27 | 20.57 |  |
| P31 | 23.45 | 20.87 | e1 | 28.55 | 24.01 |  |
| P32 | 21.78 | 19.34 | e15 | 31.79 | 27.47 |  |
| P33 | 21.47 | 19.20 | e18 | 29.67 | 25.63 |  |
| P34 | 21.76 | 20.40 | e19 | 30.80 | 26.29 |  |
| P35 | 20.91 | 18.53 | e21 | 27.92 | 23.20 |  |
| P36 | 21.78 | 21.38 | e24 | 32.45 | 27.92 |  |
| P37 | 25.14 | 25.61 |  |  |  |  |
| P39 | 23.25 | 20.52 |  |  |  |  |
| P40 | 27.66 | 25.16 |  |  |  |  |
| P41 | 21.93 | 19.55 |  |  |  |  |
| P42 | 28.12 | 26.29 |  |  |  |  |
| P43 | 31.68 | 26.43 |  |  |  |  |
| P44 | 22.32 | 19.56 |  |  |  |  |
