# Supplementary material for: Detection of erbB2 copy number variations in plasma of patients with esophageal carcinoma
Source: BMC Cancer. 2011 Apr 11;11:126. doi: 10.1186/1471-2407-11-126 (PMC3094322; doi:10.1186/1471-2407-11-126)
Supplement: Additional File 4 — Supplemental Table S3: Copy number variation of CTCs from EC patients compared to copy number variation of DNA from plasma of the same patients. [file 1471-2407-11-126-S4.DOC]

|  |  | |  | | |  | | |  | | |  | | |  | |
| --- | --- | --- | --- | --- | --- | --- | --- | --- | --- | --- | --- | --- | --- | --- | --- | --- |
|  | **Additional file 4: Supplemental Table S3.** Copy number variation (CN) of CTCs from EC patients compared to copy number variation (CN) of DNA from plasma of the same patients | | | | | | | | | | | |  | |  | |
|  | **CN patients code** | | | **Copy number variation** | | | **CTCs patients code** | | | **Copy number variation** | | |  | |  | |
|  | P2 | | | 15 | | | CTCs 2 | | | 11 | | |  | |  | |
|  | P5 | | | 3 | | | CTCs 5 | | | 4 | | |  | |  | |
|  | P15 | | | 5 | | | CTCs 15 | | | 5 | | |  | |  | |
|  | P19 | | | 5 | | | CTCs 19 | | | 5 | | |  | |  | |
|  | P22 | | | 10 | | | CTCs 22 | | | 10 | | |  | |  | |
|  | P24 | | | 11 | | | CTCs 24 | | | 9 | | |  | |  | |
|  |  | |  | | |  | | |  | | |  | | |  | |
|  | |  | | |  | | |  | | |  | | |  | |  |

* The patients code refers to DNA derived from plasma

and CTCs refers to DNA extracted from circulating

tumors cells of the same patients.
