# Supplementary material for: Detection of erbB2 copy number variations in plasma of patients with esophageal carcinoma
Source: BMC Cancer. 2011 Apr 11;11:126. doi: 10.1186/1471-2407-11-126 (PMC3094322; doi:10.1186/1471-2407-11-126)
Supplement: Additional File 6 — Supplemental Table S4: Quantization of DNA extracted from plasma of patients and controls. [file 1471-2407-11-126-S6.DOC]

**Additional file 6: Supplemental Table S4.** Quantization of DNA extracted from plasma of

patients and controls.

| ***CN patients code*** | ***DNA from plasma concentration (ng/μl)*** | ***Healthy control subjects code*** | ***DNA from plasma concentration (ng/μl)*** |  |
| --- | --- | --- | --- | --- |
| P1 | 144 | e10 | 40 |  |
| P2 | 159 | e12 | 34 |  |
| P3 | 144 | e13 | 27 |  |
| P4 | 95 | e14 | 14 |  |
| P5 | 239 | e2 | 7 |  |
| P6 | 201 | e20 | 78 |  |
| P7 | 178 | e22 | 64 |  |
| P8 | 197 | e23 | 8 |  |
| P9 | 265 | e3 | 33 |  |
| P10 | 155 | e40 | 45 |  |
| P11 | 137 | e41 | 67 |  |
| P12 | 298 | e46 | 86 |  |
| P13 | 301 | e48 | 53 |  |
| P14 | 164 | e49 | 37 |  |
| P15 | 234 | e5 | 10 |  |
| P16 | 209 | e51 | 24 |  |
| P17 | 309 | e52 | 39 |  |
| P18 | 258 | e53 | 28 |  |
| P19 | 123 | e55 | 20 |  |
| P20 | 143 | e56 | 7 |  |
| P22 | 276 | e57 | 81 |  |
| P24 | 302 | e6 | 90 |  |
| P25 | 175 | e7 | 38 |  |
| P26 | 274 | e8 | 66 |  |
| P27 | 128 | f3 | 89 |  |
| P28 | 132 | f4 | 36 |  |
| P29 | 298 | f5 | 73 |  |
| P30 | 304 | f6 | 82 |  |
| P31 | 309 | e1 | 94 |  |
| P32 | 259 | e15 | 13 |  |
| P33 | 155 | e18 | 56 |  |
| P34 | 235 | e19 | 58 |  |
| P35 | 354 | e21 | 83 |  |
| P36 | 125 | e24 | 27 |  |
| P37 | 249 |  |  |  |
| P39 | 187 |  |  |  |
| P40 | 299 |  |  |  |
| P41 | 310 |  |  |  |
| P42 | 322 |  |  |  |
| P43 | 182 |  |  |  |
| P44 | 221 |  |  |  |
|  |  |  |  |  |
